# Protein Kinase D1 Signaling in Cancer Stem Cells with Epithelial-Mesenchymal Plasticity

**DOI:** 10.3390/cells11233885

**Published:** 2022-12-01

**Authors:** Yichen Guo, Yinan Jiang, J. Bart Rose, Ganji Purnachandra Nagaraju, Renata Jaskula-Sztul, Anita B. Hjelmeland, Adam W. Beck, Herbert Chen, Bin Ren

**Affiliations:** 1 Department of Surgery, Heersink School of Medicine, University of Alabama at Birmingham, Birmingham, AL 35294, USA; 2 O’Neal Comprehensive Cancer Center, Heersink School of Medicine, University of Alabama at Birmingham, Birmingham, AL 35294, USA; 3 Department of Medicine, Division of Hematology and Oncology, Heersink School of Medicine, University of Alabama at Birmingham, Birmingham, AL 35294, USA; 4 Department of Cell Developmental and Integrative Biology, Heersink School of Medicine, University of Alabama at Birmingham, Birmingham, AL 35294, USA; 5 GBS Biomedical Engineering Program, Graduate School, University of Alabama at Birmingham, Birmingham, AL 35294, USA

**Keywords:** cancer stem cells, CD36, E-cadherin, epithelial to mesenchymal transition (EMT), lysophosphatidic acid (LPA), pancreatic neuroendocrine tumors, protein kinase D, vimentin

## Abstract

Pancreatic neuroendocrine tumors (pNETs) are extremely diverse and highly vascularized neoplasms that arise from endocrine cells in the pancreas. The pNETs harbor a subpopulation of stem cell-like malignant cells, known as cancer stem cells (CSCs), which contribute to intratumoral heterogeneity and promote tumor maintenance and recurrence. In this study, we demonstrate that CSCs in human pNETs co-express protein kinase PKD1 and CD44. We further identify PKD1 signaling as a critical pathway in the control of CSC maintenance in pNET cells. PKD1 signaling regulates the expression of a CSC- and EMT-related gene signature and promotes CSC self-renewal, likely leading to the preservation of a subpopulation of CSCs at an intermediate EMT state. This suggests that the PKD1 signaling pathway may be required for the development of a unique CSC phenotype with plasticity and partial EMT. Given that the signaling networks connected with CSC maintenance and EMT are complex, and extend through multiple levels of regulation, this study provides insight into signaling regulation of CSC plasticity and partial EMT in determining the fate of CSCs. Inhibition of the PKD1 pathway may facilitate the elimination of specific CSC subsets, thereby curbing tumor progression and metastasis.

## 1. Introduction

Pancreatic neuroendocrine tumors (pNETs or PanNETs) are a group of heterogeneous, extremely vascularized neoplasms that arise from endocrine cells in the pancreas [1,2,3,4]. These highly angiogenic tumors demonstrate resistance to antiangiogenic therapy and even exhibited malignant progression after antiangiogenic treatment in animal models [1,2,4,5,6]. They also elevate expressions of vascular endothelial growth factor receptors (VEGFRs) and their ligands (VEGFs) [7], which are key factors in the regulation of angiogenesis [8,9]. Targeted therapies against the VEGF signaling pathway have been approved in the treatment of pNET patients, including those with unresectable and metastatic disease, but demonstrate limited therapeutic efficacy, due to the potential for increased invasion and high rates of metastases [4,10,11,12].

Cancer stem (CS)-like cells (CSCs) contribute to phenotypic changes in endothelial cells (data not shown) and tumor-associated angiogenesis [13]. Considering the intratumoral heterogeneity of malignant cells associated with CSC development, and the potential value of targeting the vascular niches in CSCs [14,15,16,17], understanding the mechanisms by which CSCs are regulated provides insights into the development of potential anti-cancer therapies. In a variety of malignant tumors, CSCs are able to self-renew, and have higher malignancy potential, thereby promoting neoplastic maintenance, heterogeneity, and metastasis [14,16,18,19]. Studies showed that CSCs also exist in pNETs and other solid neuroendocrine tumors [20,21]. Despite its key role in CSC maintenance and tumor progression, little is known about the regulation of CSCs in pNETs. Understanding the mechanisms of CSC maintenance in the pNETs has proven challenging but is critical for the development of novel therapeutic approaches against advanced pNETs.

Many signaling pathways, including chromatin remodeling, Notch1 and PI3K/AKT/mTOR signaling, may be central to the genetic heterogeneity of pNETs. These pathways may also be vital for the control of CSC functions and pNET progression [4,5,16,22]. However, the signaling mechanisms by which pNET CSCs are regulated during tumor maintenance or progression is largely unknown. Protein kinase D1 (PKD1/PKD-1), a member of the serine/threonine kinase D family, is involved in the regulation of chromatin remodeling by modulating histone deacetylases [23,24]. PKD1 also activates PI3K/Akt signaling and regulates Notch 1 signaling in several different types of cells, such as cancer cells and vascular endothelial cells [8,17,25,26,27,28,29,30]. Among many biological outcomes of PKD1 signaling are increased angiogenesis and tumor progression [8,26]. The PKD family contains three isoforms, including PKD1, 2 and 3 [8], which can be activated by lysophosphatidic acid (LPA) [27,31], a lipid signaling mediator that can promote stem cell-like features in cancer cells and malignant progression [17,32,33,34,35,36]. However, PKD1 is predominantly expressed in pNET cells, where it regulates hormone secretion [37]. A recent study also indicated that this kinase initiates acinar cell progression and reprogramming to intraepithelial neoplasia [38].

In addition to the regulation of a variety of cancer-associated biological processes [26], PKD1 signaling actually promotes CSC maintenance in some cancers [17,39]. Activation of the PKD1 pathway is also critical to upregulating SC markers and generating a unique population of pancreatic CSCs [40]. However, this pathway has not been well explored in pNET CSCs. We intended to investigate the role of PKD1 signaling in the maintenance of pNET CSCs. Unexpectedly, we identified pNET cells that presented stem-like features with cellular plasticity when these cells were exposed to LPA, a phenotype that has long been recognized in stem cell populations [41]. They were likely undergoing partial epithelial to mesenchymal transition (EMT). The EMT is known as a cellular transdifferentiation program that enables epithelial cancer cells to become invasive and metastatic [42,43] and stem cell-like [42]. The results from this study suggest that PKD1 signaling may endow pNET cells with unique plasticity and potential partial EMT traits; thereby, enabling them to maintain flexible CSC states rather than terminal EMT states. Ultimately, these changes in phenotype could facilitate tumor initiation and completion of the metastatic processes during pNET progression.

## 2. Materials and Methods

### 2.1. Key Reagents and Antibodies

Oleoyl-L-α-lysophosphatidic acid (LPA, L7260) and a Periodic Acid-Schiff (PAS) Kit (395B) were purchased from Sigma-Aldrich. The PKD inhibitor CRT0066101 (A8679) was purchased from (APExBio, Boston, MA, USA). RNeasy Kit (Qiagen, Hilden, Germany), SYBR Green and cDNA synthesis Kits (Applied Biosystems, Waltham, MA, USA) used for transcript expression levels. Primers for GAPDH and target genes were purchased from Integrated DNA Technologies (IDT, Redwood City, CA, USA and Qiagen, Hilden, Germany) DAPI with Mounting Medium (H-1200) was procured from Vector Laboratories (Newark, CA, USA). A DAB substrate kit (8059) was purchased from CS Technology (New York, NY, USA). Opti-MEM I (51985-034) was obtained from Gibco (Hilden, Germany). The detailed information of primary antibodies used for Western blot (WB), immunofluorescence (IF), and immunohistochemistry (IHC) are provided in Supplementary Table S1.

### 2.2. Cell Culture

Human pancreatic NET cell lines BON (BON, provided by Dr. Mark Hellmich, The University of Texas Medical Branch at Galveston) and QGP-1 (obtained from the Japanese Collection of Research Bioresources Cell Bank (JCRBC)) were cultured, according to our routine and standard protocol. Briefly, BON or QGP-1 cells were, respectively, grown in glutamine-containing DMEM: F-12 media (Gibco) or RPMI1640 (Corning, Lawrenceville, GA, USA) media with 10% fetal bovine serum (FBS), containing penicillin/streptomycin, at 37 °C and in the presence of humidity and 5% CO_2_.

### 2.3. Real Time RT-qPCR

The mRNA levels were assayed as described below. Briefly. RNA was extracted from tumor cells by using the RNeasy Kit. Then, cDNA synthesis and messenger levels were determined using RT-qPCR (Bio-Rad, Philadelphia, PA, USA). GAPDH mRNA were amplified in distinct wells for targeted messenger normalization. Ct value was employed to normalize and quantify changes in transcript levels.

### 2.4. Immunoblot Assays

BON and QGP-1 cell line extracts were collected by processing with RIPA buffer (Sigma), and protein levels were quantified using a BCA kit (Pierce Chemical, Dallas, TX, USA). Cell lysates were collected to run on polyacrylamide gel, and transferred to PVDF membranes, followed by immunoblotting with appropriate antibodies. NIH Image J (https://imagej.nih.gov/ij/index.html, accessed on 17 October 2022) was used for densitometry to determine relative expression of target proteins.

### 2.5. Human pNET Specimens

Immunofluorescence and immunohistochemistry were performed for pNET patient specimens. The investigators did not know information about the patients [44]. The specimens included tissue samples from four pNET patients, a tissue microarray (TMA) slide generated with pNET tissues samples from 35 patients, and a control TMA slide of human organs from 33 normal individuals (UAB Department of Pathology).

### 2.6. Immunofluorescence and Immunohistochemistry

Briefly, paraffin tissues were deparaffinized and dehydrated using Histo-Clear and a gradient of ethanol washes, respectively. Antigen retrieval was done by immersing slides in antigen unmasking solutions (Vector 21202), placing the slides in a microwave oven for 1 min and boiling the slides at 95–99 °C for 15 min. The sections were cooled to 22 °C (RT) and washed with distilled water and PBS. Then, 5% BSA was added to block non-specific reactions. The slices were incubated with indicated antibodies, followed by appropriate secondary antibodies. The slices were exposed with DAB chromogen, hematoxylin, and Periodic Acid-Schiff (PAS), based on the manufacturer’s instructions. The images were captured on an Olympus microscope or an All-in-One Fluorescence Microscope BZ-X810 (Keyence, Itasca, IL, USA).

For Immunofluorescence, briefly, pNETs cell lines were fixed with 4% PFA, and permeabilized in 0.3% Triton-100. Then, the cell samples were blocked in 3% BSA for 1 h and probed with appropriate primary and secondary antibodies. The immunofluorescence images were captured on an All-in-One Fluorescence Microscope BZ-X810 (Keyence).

### 2.7. Aldehyde Dehydrogenase (ALDH) Activity Assays

The ALDH1 ELISA kit (ab155894, Abcam, Cambridge, MA, USA) was used to measure ALDH1 activity in BON and QGP-1 cells with different treatments. The reaction mixture of ALDH assay buffer and ALDH substrate was incubated for 60 min at 22 °C. Fluorescence values were recorded at Ex/Em 535/587 nm by the Gen5 program (BioTek, Winooski, VT, USA) and used to calculate ALDH activity, based upon the manufacturer’s instruction.

### 2.8. Tumorsphere Formation Assays

An amount of 5000 cells were cultured into each well of an ultra-low attached 6-well plate in 2 mL complete MammoCultTM Medium (Stem Cell Technologies, Vancouver, BC, Canada), based upon the manufacturer’s instructions. In some experiments, tumorspheres were exposed to vehicle control or reagents every three days. The numbers of tumorspheres were randomly counted with eight repetitions after culture for 7 days. Images of tumorspheres were captured on an OLYMPUS LH50A microscope (Feasterville, PA, USA).

### 2.9. In Vitro Extreme Limiting Dilution and Tumorsphere Formation Assays

BON cells were seeded with a decreasing number of cells per well in an ultra-low attached 96-well plate. The tumorspheres were cultured in MammoCult™ Medium. The number of tumorspheres was counted after 7 days of culture under stem cell culture conditions. The data was evaluated and the log-fraction figure made accordingly, using software available at bioinf.wehi.edu.au/software/elda/.

### 2.10. Statistics

Quantitative data are presented as mean ± SD or mean ± SEM. Data were analyzed using 2-sided unpaired *t* tests or one-way ANOVA using a GraphPad software package Prism 9. A *p* < 0.05 or 0.01 was considered statistically significant or very significant.

## 3. Results

### 3.1. Regulation of Stem-like Phenotype in pNETs by PKD1 Signaling

**Figure 1:**
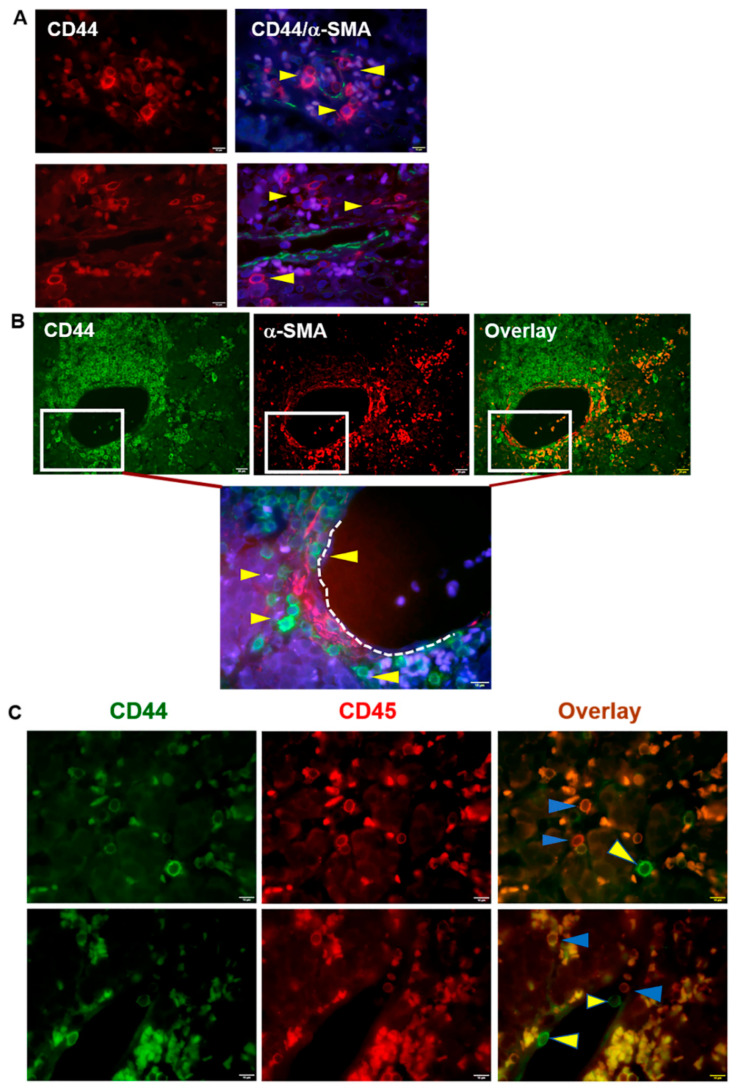
Identification and distribution of CD44-positive cancer stem-like cells in human pNET tissues. (**A**) Human pNET specimens in patient 1 were co-stained with CD44 and α-SMA antibodies, followed by appropriate secondary antibodies, with DAPI staining the nuclei (blue). Images were acquired by immunofluorescence microscopy. CD44-positive cancer cells (red) marked by yellow arrowheads were close to the α-SMA positive (green) blood vessels. (**B**) Human pNET specimens in patient 2 were co-stained as in (**A**). CD44-positive cancer cells (green) were indicated by yellow arrowheads, which were close to the α-SMA positive (red) blood vessels. A subset of CD44-positive cells appeared to be distributed within the α-SMA-positive vascular niche or nearby blood vessels. Shown are representative images from individual patients. Bar = 10 or 20 µm. (**C**) Human pNET tissues were co-stained with CD44 antibodies and CD45 antibodies followed by appropriate secondary antibodies. Overlay images were collected by immunofluorescence microscopy. CD44-positive (green, yellow arrowheads) and both CD44-positive and CD45-positive cells (orange, blue arrowheads) were observed under a fluorescence microscope. Images were acquired by an Olympus BX60 fluorescence microscope equipped with a CCD camera. Shown are representative images from two individual patients. Bar = 10 µm. Cells positive with both CD44 and CD45 are smaller in size and nucleus, and regarded as lymphatic cells (orange), a subset of cancer cells, which are bigger and positive for CD44 but negative for CD45, suggesting their stem cell-like phenotype.

CSCs are a subpopulation of cells within a tumor that are able to initiate tumors when propagated in animal models, sustain proliferation, and promote metastasis and therapeutic resistance [16,18,19,45,46,47]. There are CSCs and aggressive cancer cells in close proximity to blood vessels as the perivascular niche enables perfusion of oxygen and nutrients to nearby cells [17,48]. To determine and identify the presence and distribution of stem-like cancer cells in pNETs, we initially stained tissue sections from human pNET patients with the CSC marker CD44, the pericyte/vascular smooth muscle cell marker α-SMA, and the lymphatic cell marker CD45 that can differentiate CD44^+^/CD45^−^ CSCs from non-stem-like CD44^+^/CD45^+^ lymphatic cells. Immunofluorescence microscopy showed that groups of CD44-positive pNET cells were adjacent to vascular networks within the tumor microenvironment. In contrast, the glandular epithelial cells in normal pancreatic tissue expressed minimal levels of CD44 (Figure 1A,B and Figure S1). Similar to our previous study on breast cancers [17], a subset of a few CD44^+^/CD45^−^ tumor cells detached from their nests and accumulated near the capillaries or around the α-SMA^+^ arterioles, along with the presence of more CD44^+^/CD45^+^ lymphatic cells (Figure 1C).

**Figure 2:**
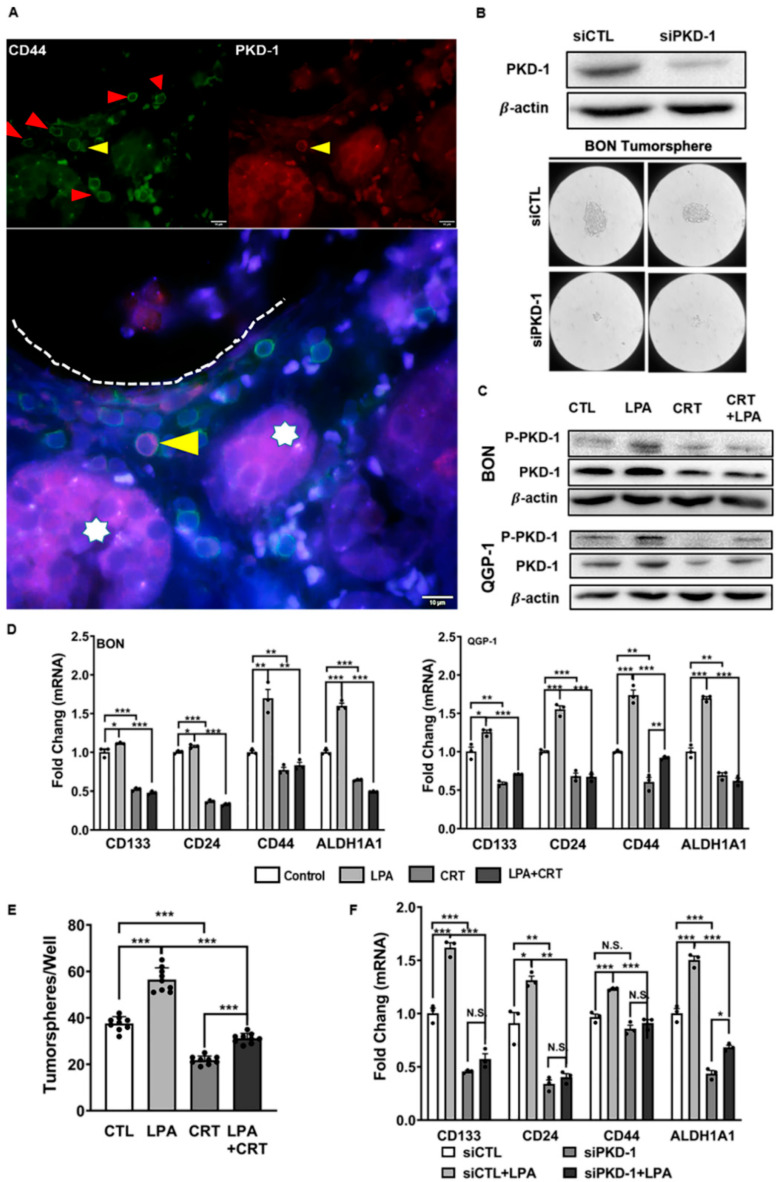
PKD1 signaling in the maintenance of cancer stem-like features in pNETs. (**A**) Distribution of PKD1^+^ and CD44^+^ CSCs within the vascular niche. Human pNET specimens were co-stained with CD44 and PKD1 antibodies, followed by appropriate secondary antibodies, with DAPI staining the nuclei (Blue). Stem-like cells with CD44-positive (green), PKD1-positive (red) or both positive (pink) were observed under a fluorescence microscope. A few CD44-positive cancer stem-like cells tended to accumulate near the vascular lumen (red arrow heads). Cancer cell with moderate expression of both PKD1 and CD44 might be leaving tumor nests (stars) for the vascular lumen. The fluorescence images were acquired by a fluorescence microscope equipped with a CCD camera. Shown are representative images. Scale bar = 10 µm. (**B**) BON cells were transfected with siRNA control and siPKD1 to knock down PKD1. Knockdown efficiency was confirmed by Western Blots (upper panel). The control and BON cells with PKD1 knockdown were subjected to tumorsphere formation assays. Images were acquired by the OLYMPUS CK30 microscope. Representative images are shown for tumorsphere formation (lower panel). Scale bar = 200 µm. (**C**) Cell lysates were extracted from BON and QGP-1 cells exposed to the vehicle control, 10 μM LPA, 2 μM CRT0066101, or their combinations after 24 h. The expression levels of phosphorylated PKD1 and total PKD1 were detected by Western blots. Shown are representative images of triplicate experiments in BON (upper panel) and QGP-1 (lower panel) cells. (**D**) BON and QGP-1 cells were exposed to 10 μM LPA, 2 μM CRT0066101, or their combination for 24 h, and total RNA was extracted for the detection of mRNA levels of genes related to stemness properties by RT-qPCR. (**E**) Effect of PKD inhibitor in tumorsphere formation. BON cells were cultured in complete MammoCult™ medium with the treatment of 10 μM LPA, 2 μM CRT0066101, or their combination for 7 days. The number of mammary spheres was counted under the OLYMPUS CK30 microscope. (**F**) Control and BON cells with PKD1 knockdown were exposed to 10 μM LPA, 2 μM CRT0066101, or their combination for 24 h, and total RNA was extracted for the detection of mRNA levels of genes related to stemness properties by RT-qPCR. Triplicate experiments were performed, and the results are shown as the mean ± SEM. * p < 0.05, ** p < 0.01, and *** p < 0.001.

To determine if the PKD1 pathway was associated with CSCs in pNETs, we further observed the expression of PKD1 and CD44 in tumor tissues from human pNET patients. Interestingly, tumor cells in cancer nests showed low to moderate levels of PKD1 expression, along with low levels of CD44 expression. In contrast, disseminated tumor cells detached from the tumor nests and invaded stroma and nearby blood vessels. They demonstrated relatively high levels of CD44 and PKD1. The elevation of PKD1 and CD44 was particularly high in tumor cells close to the blood vessels (Figure 2A and Figure S2). To clarify the function of PKD1 in the preservation of a stem cell-like state in pNETs, we genetically targeted PKD1 in BON cells. As presented in Figure 2B, transfection of siRNA significantly reduced the expression of endogenous PKD1. Concomitantly, tumorsphere formation capacity was impaired in these tumor cells with PKD1 knockdown (Figure 2B).

Lysophosphatidic acid (LPA), a lipid signaling mediator, activates PKD1 and promotes tumor initiation, the development of CSC-like features and metastasis [17,32,33,34,35,36]. The presence of autotaxin, a key enzyme in producing LPA in the primary metastatic subtype of pNETs, suggests that LPA signaling may be associated with the metastatic potential of pNETs [3]. To determine the function of PKD1 signaling in CSC maintenance, we treated pNET cells with LPA to induce CSC features. As shown in Figure 2C and Figure S3A,B, pNET cells exposed to LPA activated the PKD1 signaling pathway, and the pathway was effectively targeted with a PKD inhibitor CRT0066101. Moreover, LPA treatment in the cancer cells stimulated the CSC-related gene signature, via the PKD signaling (Figure 2D), including CD133 and CD44, two common CSC markers, whose expressions are connected with a poor prediction in pNET patients [49]. Furthermore, pharmacological inhibition of LPA-induced PKD1 signaling, using the PKD inhibitor, attenuated tumorsphere formation (Figure 2E), a hallmark of CSC-like cells [50]. To further confirm the essential function of PKD1 signaling, rather than PKD2 or PKD3 isoforms, in the control of stem cell-like features, we knocked down endogenous PKD1 expression in BON cells to examine expression of the stemness-related gene signature. LPA treatment significantly increased mRNA expression of CD44, CD133, CD24, and ALDH1A1. However, there was a noticeable decrease in LPA-induced expression of these CSC-related genes with PKD1 knockdown (Figure 2F).

**Figure 3:**
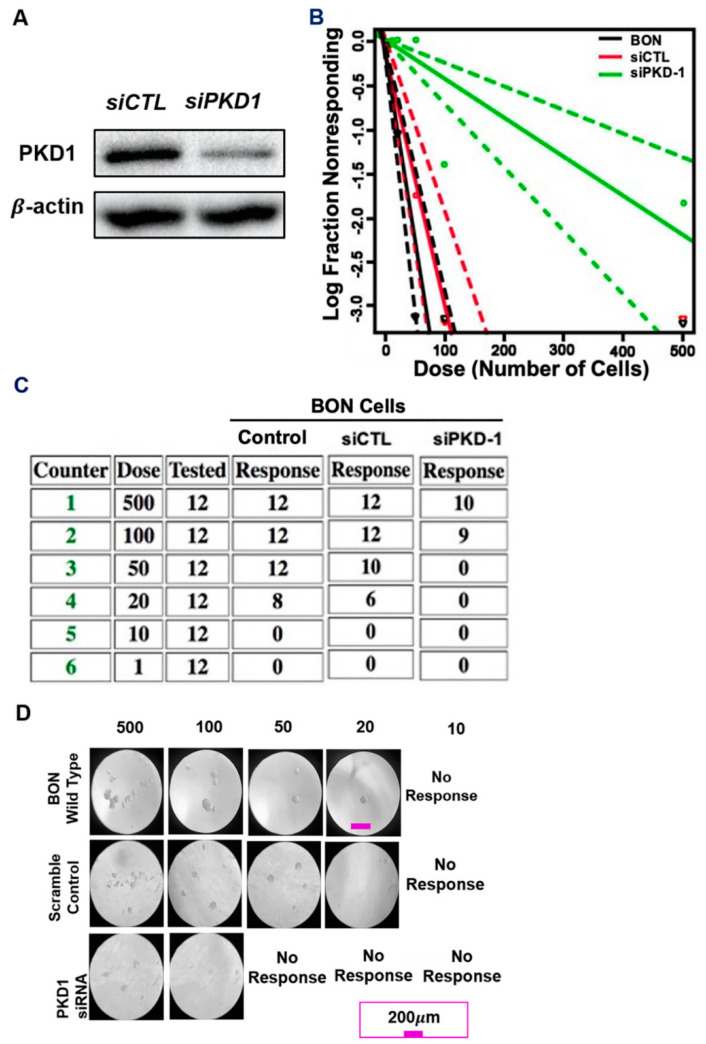
Requirement of PKD1 signaling in CSC maintenance and potential tumorigenicity. (**A**) Immunoblotting assay indicated that transfection of siPKD1 resulted in a significant PKD1 downregulation in BON cells, as compared with regular BON cell control (CTL) and the scramble control (siCTL). (**B**) The log fraction nonresponding figure showed that tumor formation efficiency of PKD1 deficient BON cells decreased, compared with the regular and scramble control BON cells, in the tumorsphere formation efficiency assays. (**C**) Limiting dilution tumorsphere formation efficiency assay data showed the number of tumorsphere formations of control, scramble control and PKD1-depleted BON cells among 12 wells with the seeding densities starting from 500 cells/well to 1 cell/well. (**D**) Representative images for tumorsphere formation in regular BON cell control, scramble cell control and PKD1 knockdown BON cells with different seeding densities. Scale bar = 200 µm.

To further validate PKD1 signaling in the maintenance CSC traits in pNETs, we performed a well-established, and most widely accepted, in vitro limiting dilution tumor assay to assess the impact of this molecule in the regulation of tumor-initiating potential, a key functional feature in CSCs. As shown in Figure 3A, transfection of siRNA demonstrated an efficient knockdown of endogenous PKD1 expression at the protein level. We then examined the number of tumorspheres and observed obvious tumorsphere formation capacity, with a seeding density ranging from 500 to 20 cells/well. As shown in a log fraction nonresponding figure, there was a significant decrease of tumor formation efficiency (TFE) in PKD1-knockdown BON cells (Figure 3B). Similar to a previous study in breast cancer cells [17], PKD1 knockdown significantly compromised the frequency of repopulation of the cancer stem-like cells, when compared with the control pNET cells (Figure 3B–D). These results suggest that PKD1 signaling may play an essential role in the maintenance and expansion of CSCs in pNETs, as well as having tumor initiation capacity.

### 3.2. Requirement of PKD1 Signaling in Partial EMT and CSC Plasticity

**Figure 4:**
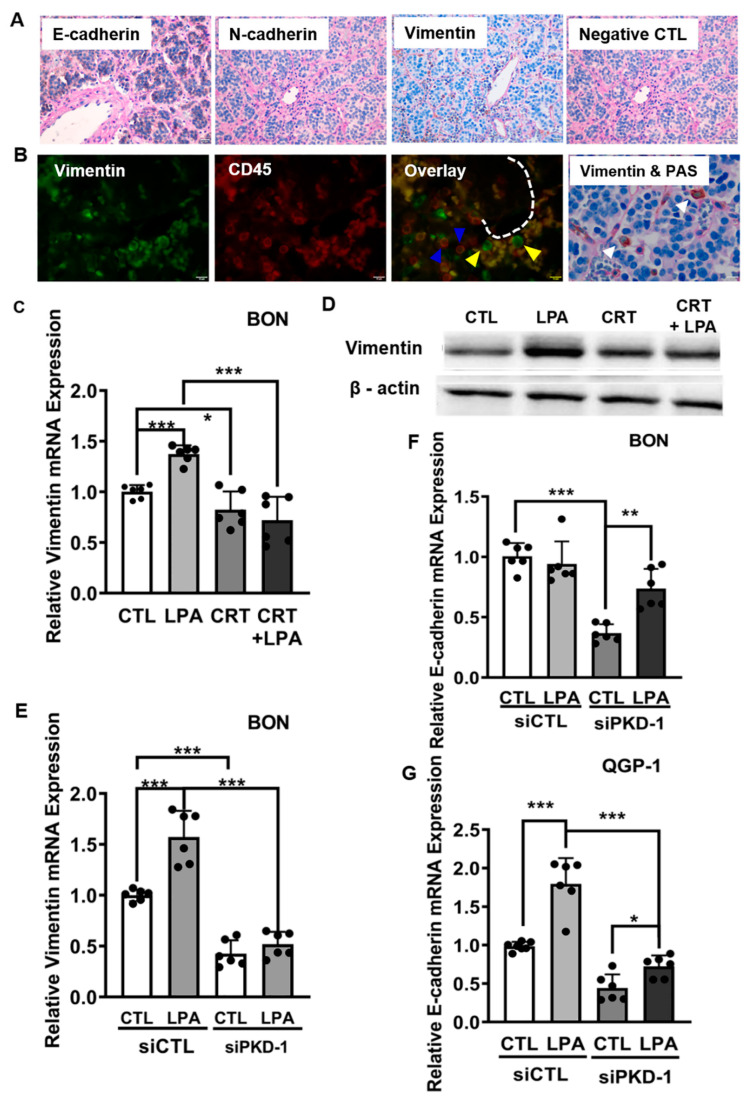
Regulation of EMT by PKD1 signaling in pNETs. (**A**) Human pNET tissues were stained with E-cadherin, N-cadherin and vimentin antibodies by immunohistochemistry (IHC), along with staining by Periodic Acid-Schiff (PAS) for the matrix. Tumor cells expressed E-cadherin but little N-cadherin. A small subset of tumor cells, that detached from a cancer nest and distributed in the vascular network, expressed mesenchymal marker vimentin. Bar = 20 µm. (**B**) Human pNET tissues were co-stained by vimentin and CD45 antibodies, followed by appropriate secondary antibodies. Vimentin-positive (green) and/or CD45-positive (red) cells were observed under an immunofluorescence microscope. The fluorescence images were acquired by an immunofluorescence microscope equipped with a CCD camera. Shown are representative images. Bar = 10 µm. Vimentin-positive and CD45-negative tumor cells (yellow arrowhead) were mainly located within the vascular network; vimentin-positive CD45-positive lymphatic cells are indicated by blue arrowhead. Double-staining with IHC and PAS showed that these vimentin-positive mesenchymal tumor cells (white arrowhead) were located in the vascular network or detached from their nests, and exhibited larger nuclei, compared to lymphatic cells (lower panel right). Vimentin antigens were present, indicated as brown color by IHC (HRP-DAB), and vascular basement membrane is shown as pink by PAS-staining. Bar = 10 µm. (**C**) BON cells were treated with 10 µM LPA, 2 µM CRT0066101, or their combination in serum-free medium for 24 h. Total RNA was extracted from each group to assay mRNA levels of vimentin by RT-qPCR. (**D**) BON cells were cultured in DMEM/F12 medium with 5% FBS. After starvation in serum-free DMEM/F12 medium for 6 h, the cells were treated with 10 µM of LPA, and/or 5 µM of CRT in serum-free DMEM/F12 medium for an additional 24 h under 5% CO_2_ at 37 °C. Cell lysates were collected and subjected to Western blots for vimentin expression. Shown is a representative image. (**E**) BON cells were transfected with a scramble control or PKD1 siRNA for 24 h, followed by treatment with 10 µM LPA for 24 h. Total RNA was isolated for vimentin gene expression by RT-qPCR. (**F**) BON cells were transfected and treated as (**E**) for E-cadherin gene expression by RT-qPCR. (**G**) QGP-1 cells were transfected and treated as (**E**) for E-cadherin gene expression by RT-qPCR. Triplicate experiments were performed. * *p* < 0.05, ** *p* < 0.01, *** *p* < 0.001.

EMTs function as major mechanisms in tumor invasion and metastasis [43,51,52,53] and are implicated in generating CSCs [42]. Metastatic tumor cells present different epithelial or mesenchymal phenotypes from cells in tumor nests. To determine EMT features in different subsets of tumor cells, we examined the expression of vimentin, N-cadherin, and E-cadherin in human pNET tissues, by IHC, along with Periodic Acid-Schiff (PAS) double staining for the matrix. We observed vimentin expression (mesenchymal marker) in some tumor cells (Figure 4A,B; Figure S3A for H & E staining), and these cells tended to be distributed close to the vascular network (Figure 4B) or in a manner similar to PKD1-positive CSCs (Figure 2A). Intriguingly and unexpectedly, there was also clear expression of E-cadherin in most of the tumor cells, an indicator of mesenchymal to epithelial reverting transitions during the metastatic seeding of disseminated carcinomas [54], with little expression of the mesenchymal marker, N-cadherin (Figure 4A).

LPA as a PKD1 activator regulates an EMT program in tumor progression [55]. This lipid signaling mediator may increase in a subtype of primary metastatic tumors in pNETs [3]. To define whether PKD1 signaling regulates vimentin expression or not, we exposed pNET cells to LPA, the PKD inhibitor, or their combination, and examined vimentin mRNA levels in response to LPA/PKD-1 signaling. LPA exposure increased protein expression of both phosphorylated and total PKD1 in BON cells (Figure S3B) and QGP-1 cells (Figure S3C). Intriguingly, exposure to LPA increased expression of vimentin mRNA in BON (Figure 4C) and QGP-1 cells (Figure S4A). Treatment with a PKD inhibitor prevented LPA-mediated induction of vimentin at mRNA levels (Figure 4C and Figure S4A). Immunoblotting confirmed that the PKD inhibitor repressed LPA-induced vimentin protein expression (Figure 4D). Furthermore, PKD1 knockdown reduced endogenous vimentin expression and abolished LPA-mediated upregulation of vimentin mRNA in BON (Figure 4E) and QGP-1 cells (Figure S4B). LPA treatment in BON cells did not increase E-cadherin mRNA, but PKD1 knockdown downregulated endogenous E-cadherin expression, which was partially rescued by treatment with LPA (Figure 4F). Intriguingly, LPA increased E-cadherin expression in QGP-1 cells. However, genetic targeting of PKD1 in QGP-1 cells reduced the E-cadherin mRNA levels by knocking down its endogenous expression, which could be partially rescued by LPA exposure (Figure 4G).

**Figure 5:**
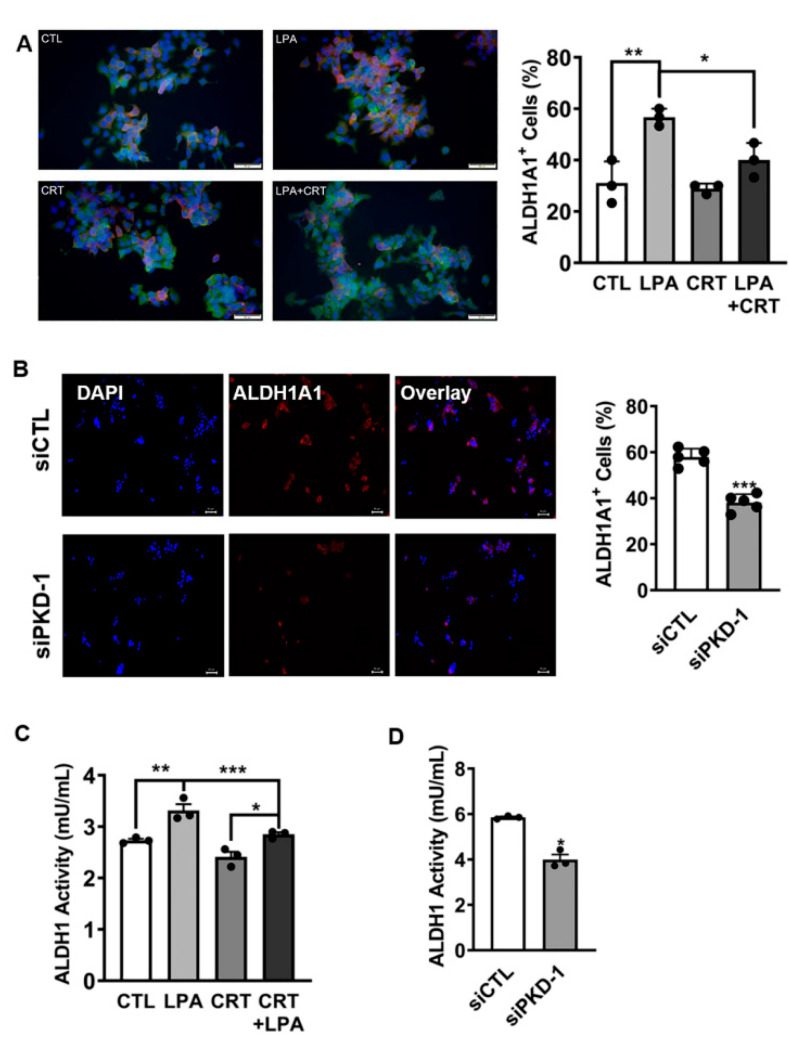
Association of PKD1 signaling with ALDH1. (**A**) BON cells were cultured in DMEM/F12 medium with 5% FBS. After starvation in serum-free DMEM/F12 medium for 6 h, the cells were treated with 10 µM of LPA, and/or 5 µM of CRT in serum free DMEM/F12 medium for an additional 24 h under 5% CO_2_ at 37 °C. The cells were incubated with ALDH1A1 and PKD1 antibodies, followed by appropriate secondary antibodies. The percentages of cells with high levels of ALDH1A1 expression (red) were calculated by randomly counting up to 30 individual cells, and triple counting was performed. GraphPad Prism 9 was used for statistical analysis. (**B**) BON cells were transfected with scramble control or PKD1 siRNA for 24 h, and the cells were processed for staining with an ALDH1A1 antibody, followed by an appropriate secondary antibody. The ratio of cells with high levels of ALDH1A1 expression were calculated under a fluorescence microscope by counting up to 100 cells randomly in each field. Five repetitions were performed. Statistic difference was evaluated by GraphPad Prism 9. (**C**) BON cells were treated with 10 µM LPA, 2 µM CRT0066101, or their combination for 24 h. ALDH1 activity was measured by ELISA in a plate reader. (**D**) BON cells were transfected with scramble control or PKD1 siRNA to knock down endogenous expression. ALDH1 activities were measured by ELISA in a plate reader. Triplicate experiments were performed. The results were shown as the mean ± SEM. * *p* < 0.05, ** *p* < 0.01, and *** *p* < 0.001.

ALDH1 activity is essential for CSC plasticity and metastatic potential [56,57]. Given that PKD1 plays a vital role in the control of ALDH1A1 transcript expression in pNET CSCs (Figure 2D), we treated BON cells with LPA, a PKD inhibitor, or their combination, and examined ALDH1A1 expression. We did not find major deviations in ALDH1A1 protein expression by immunoblotting in BON cells exposed to LPA and/or a PKD inhibitor (data not shown). However, immunofluorescence demonstrated that the percentage of cells with enhanced ALDH1A1 expression (ALDH1A1^+^) increased following LPA treatment (*p* < 0.01), and this effect was prevented by co-treatment with the PKD inhibitor (*p* < 0.05) (Figure 5A). To confirm the essential function of PKD1 on ALDH1A1 levels, we knocked down endogenous PKD1 expression by transfection. PKD1 knockdown led to a decrease in the number of ALDH1A1^+^ BON cells (Figure 5B). There was also a decrease in the number of cells with enhanced ALDH1A1 expression in QGP-1 cells by knocking down PKD1, as compared to the control (Figure S5A). Furthermore, LPA treatment moderately increased ALDH1 activity, and this increase was attenuated by co-treatment with the PKD inhibitor in both BON (Figure 5C) and QGP-1 cells (Figure S5B). In BON cells PKD1 knockdown also decreased ALDH1 activity, compared to the control (Figure 5D). Together, the results indicated that the PKD1 signaling pathway in pNET cells may be essential for the maintenance of the self-renewal capacity of CSCs with potential features of plasticity and partial EMT.

### 3.3. Critical Role of PKD1 Signaling in CD36 Expression in pNET Cells

CD36 is known as a scavenge receptor, fatty acid receptor and angiogenesis regulator [17,24,27,29,58,59]. The association of CD36 with tumorigenesis is controversial [17,29,59,60,61,62]. In some pancreatic cancers, CD36 expression is negatively associated with tumor progression [63]. However, recent studies demonstrated that CD36 drives the CSC phenotype, and increases drug resistance capacity and metastatic potential of CSCs [17,59,62,64].

**Figure 6:**
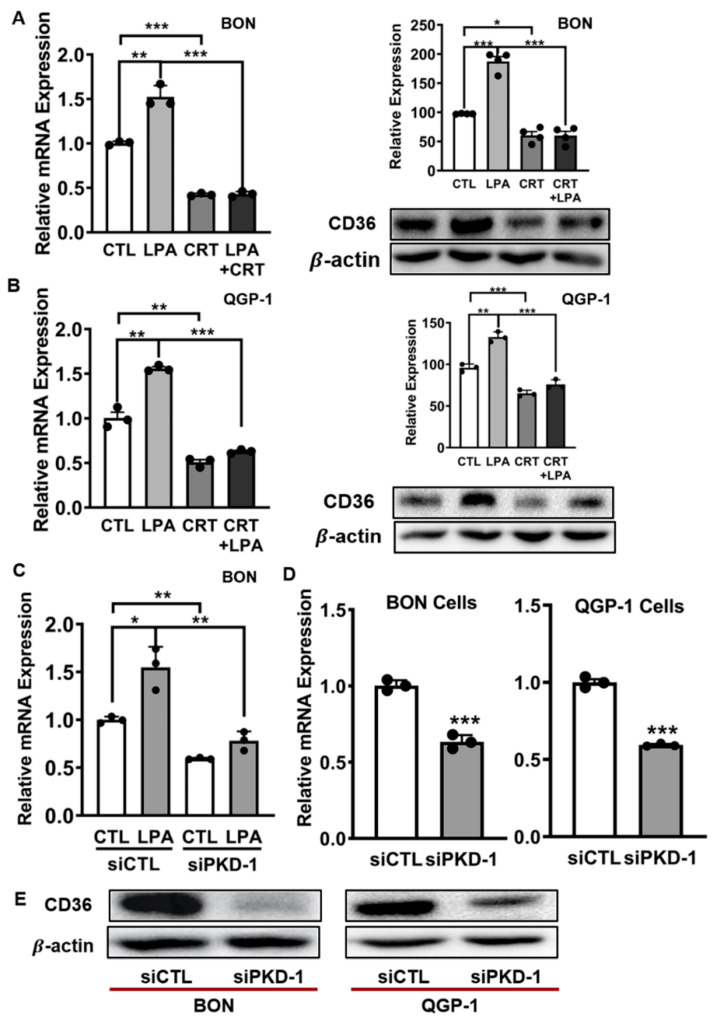
Regulation of CD36 expression by PKD1 signaling in pNET cells. (**A**) BON cells were exposed to 10 µM LPA, 2 µM CRT0066101, or their combination, for 24 h. Total RNA was isolated for CD36 gene expression by RT-qPCR (left panel), and cell lysates were collected and subjected to Western blot for CD36 protein expression (right panel). (**B**) QGP-1 cells were treated and assayed for CD36 gene and protein expression as (**A**). (**C**) BON cells were transfected with scramble control or PKD1 siRNA for 24 h, followed by treatment with 10 µM LPA for an additional 24 h. Total RNA was isolated for CD36 gene expression by RT-qPCR. (**D**) The pNET cells were transfected with scramble control or PKD1 siRNA to knock down endogenous PKD1 gene expression, and total RNA was isolated for CD36 gene expression by RT-qPCR. (**E**) The pNET cells were transfected with scramble control or PKD1 siRNA to knock down endogenous PKD1 gene expression, and cell lysates were collected and subjected to Western blotting for CD36 protein levels. Shown are representative images. CD36 protein levels were assessed by densitometry with NIH Image J. Triplicate experiments were performed. * *p* < 0.05, ** *p* < 0.01, and *** *p* < 0.001.

Since PKD1 signaling downregulates CD36 expression in vascular ECs in response to LPA treatment [17,24,27], we intended to define if PKD1 signaling regulates CD36 expression in pNETs. Toward this end, BON and QGP-1 cells were exposed to LPA and/or the PKD inhibitor. Unexpectedly, different from its role in vascular ECs, LPA treatment increased CD36 expression at both transcript and protein levels in BON (Figure 6A) and QGP-1 cells (Figure 6B). The addition of a pharmacological PKD inhibitor prevented LPA-induced CD36 expression (Figure 6A,B). To confirm the essential function of PKD1 signaling in CD36 expression, we genetically knocked down endogenous expression of PKD1. Compared with the control group, LPA stimulated mRNA expression of CD36 in BON cells, whereas PKD1 knockdown prevented the LPA-induced expression (Figure 6C). Genetic targeting of PKD1 also decreased endogenous expression of CD36 at both transcriptional (Figure 6D) and translational levels (Figure 6E) in QGP-1 and BON cells. These results suggested that PKD1 signaling could drive CD36 expression and could enhance metastatic potential and drug resistance in pNETs, via an increase of CD36-mediated fatty acid metabolism in LPA/PKD1 signaling-induced development of CSCs [36,62,64].

## 4. Discussion

CSCs that diverge in gene expression are accountable for tumor heterogeneity and drive metastasis and therapeutic resistance in a variety of cancers [16,17,18,19,59,62]. These CSCs are also present in heterogeneous pNETs [4,20,21,47,65]. This study demonstrated a subset of CSCs that were positive for CD44 and PKD1 in human pNETs. They appeared to detach from their nests and accumulated within the vascular niches, particularly in the arteriolar niche. In addition, the tissues from pNTE patients expressed both vimentin and E-cadherin. These findings need further confirmation and characterization. However, this study highlighted a critical role of PKD1 signaling in the maintenance of a subset of CSCs that demonstrated traits of plasticity and partial EMT. Mechanistically, PKD-1 signaling might regulate the expression of specific CSC- and EMT-related gene signatures, such as CD36, ALDH1A1, vimentin and E-cadherin [16,17,21,59,62,66,67,68].

Many pathways linked to oncogenesis including, Notch, Sonic hedgehog and Wnt, can regulate the self-renewal of CSCs [16,42,69,70]. Distinct pathways may control CSC self-renewal in different types of tissues. This study in pNETs demonstrated a new function of the PKD1 pathway in the control of CSC-like features with epithelial/mesenchymal plasticity and partial EMT. This result was consistent with studies indicating that EMT confers tumor-initiating and metastatic potential to cancer cells, thereby generating high-grade aggressive cells with CSC features [42,71,72]. EMT could be a key step in pNET tumorigenesis [73]. Intriguingly, this study demonstrated that, in human pNET tissues, E-cadherin was constitutively expressed, along with vimentin expression in some cells. We, thus, tried to investigate the basic molecular mechanisms. By using cellular models in pNETs, we showed that PKD1 signaling might contribute to the establishment of a partial EMT program, by inducing expression of both vimentin and E-cadherin. This PKD1-induced phenotype might be supported by the fact that MAPK/ERK and PI3K/Akt pathways, representing the downstream of PKD1 signaling, interact with a series of intracellular signaling networks to determine the actual implementation of the EMT program at cellular levels [43,74].

Vimentin is normally expressed in mesenchymal cells, while E-cadherin expression reveals an epithelial property, and ALDH1 is critical for the regulation of CSC plasticity [17,21,59,62,66,67]. This study indicated that pNET cells presented a stem cell-like plasticity, due to ALDH1A1 expression in response to LPA/PKD-1 signaling. Moreover, activation of this pathway was essential for a mixed expression of both the mesenchymal and epithelial markers in these cells.

E-cadherin is generated in most differentiated tumors [74]. Lack of E-cadherin appears to be significantly involved in EMT and tumor invasion [74,75]. However, it is reasonable to speculate that the pNET cells can maintain an invasive phenotype, despite obvious E-cadherin expression, as high levels of vimentin, ALDH1A1 and CD36 may counteract the invasion-suppressor role of the constitutively expressed E-cadherin. Meanwhile the hybrid states (epithelial-mesenchymal) of CSCs, due to co-expression of both vimentin and E-cadherin, could facilitate collective cell migration, by providing a “stemness window” rather than complete commitment toward the mesenchymal phenotype [76]. We assume that by maintaining a partial EMT phenotype or hybrid state that lies between the epithelial and the mesenchymal state, these cancer cells can show better CSC plasticity, particularly in the presence of ALDH1 expression that is critical for CSC plasticity. Furthermore, along with CD36 expression, the metastatic potential could increase significantly, due to CD36-mediated fatty acid metabolism [17,57,62,77,78]. This hybrid cell state, with specific gene signatures and mixed epithelial-mesenchymal phenotypes with retention of certain epithelial traits, might be central to the functionality of CSCs and the acquirement of more invasive capacities in tumor progression and metastasis. It should be noted that this PKD1 signaling-mediated phenotype in pNETs is different from other types of tumors, where this pathway was considered to maintain the epithelial phenotype [79]. This warrants further exploration and investigation.

This study also suggested that, in the initial stage, PKD1 signaling might promote the clustering of E-cadherin in pNET cells. This is different from other cancer types, where E-cadherin is typically repressed during EMT, and those cancer cells cannot undergo collective movement, due to E-cadherin deficiency [42]. Conversely, E-cadherin-mediated clusters of cells in pNETs may undergo malignant progression and collective dissemination [68,80,81], due to the simultaneous of both E-cadherin and vimentin. This migratory behavior may be enhanced following concomitant expression of ALDH1A1 and CD36 expression induced by environmental factors, such as rich LPA present within the tumor microenvironment [3,27]. Moreover, these factors may regulate tumor cell–microenvironment interaction, thereby promoting partial EMT and CSC maintenance [17,33,35,55]. To further support the role of LPA in pNET progression, a seminal study, using transcriptome profiling analyses in a RIP1-RT2 transgenic mouse model, identified a small number of genes that differentiate the metastasis-like primary (MLP) subtype from insulinoma tumors (IT). Interestingly, among these genes, Enpp2 (autotaxin, ATX) expression demonstrated a higher level in MLP tumors than in IT [3]. ATX is a secreted enzyme, essential for the generation of signaling lipid LPA. This study, thus, suggested that ATX might not only serve as a marker for malignant progression [3], but might also promote LPA production in the MLP subtype, which might subsequently induce the development of metastatic CSCs for metastatic progression. The ATX-LPA axis and/or LPA are generally considered to be important targets for cancer and are critical new players in CSCs [82]. It is warranted for deep mechanistic investigation as to how PKD1 signaling is involved in the ATX–LPA axis in the regulation of unique plastic CSC subsets with partial EMT in pNETs and other types of cancer with robust angiogenesis.

**Figure 7:**
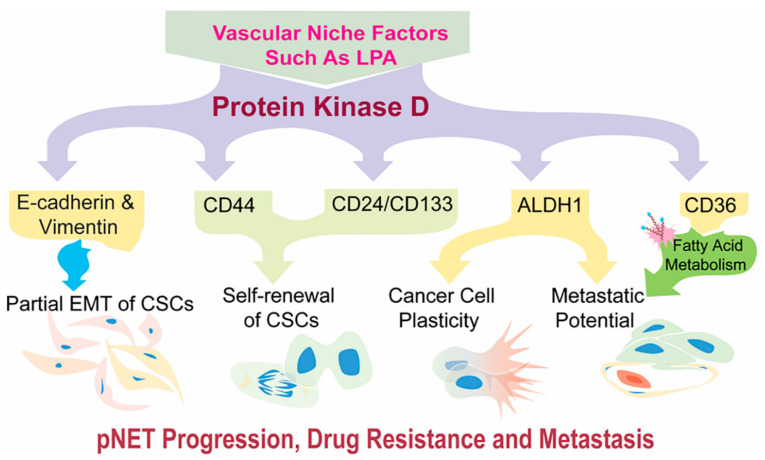
Working model: Regulation of CSCs with a plastic and partial EMT phenotype by PKD1 signaling in pNETs. PKD1 signaling may induce intermediate, or partial, EMT with a plastic phenotype in cancer stem-like cells, thereby endowing pNETs with robust invasive and pro-metastatic traits and drug resistance by regulation of different sets of stemness- and EMT-related gene expressions. Vascular niche factors, such as LPA, may contribute to the development of this phenotype in pNETs.

On the other hand, E-cadherin-positive cells could easily revert to the epithelial state during metastatic dissemination by undergoing mesenchymal–epithelial reverting transitions (MErT), due to their cellular plasticity [54]; thereby, for example, enabling pNET cells to establish secondary colonies in the liver, the most frequent organ for pNET spread. Additionally, by stimulating stemness-like features, the PKD1 signaling pathway might significantly contribute to aggressive and metastatic behavior in pNETs. Finally, this study validated previous results [17] indicating that pNET CSCs could move toward the vascular niches, in which vascular ECs are a key player. Given the critical role of ECs in arteriolar differentiation and tumor progression [15,16,17,83,84,85], vascular ECs in highly vascularized pNETs may nurture CSCs by direct EC-CSC interactions and by indirect generation of such vascular niche factors as LPA to activate PKD1 signaling [4,16,17,24,28], thereby, leading to progression toward malignancy, drug resistance and metastasis. Based upon previous studies and this study, we propose a working model (Figure 7).

In summary, PKD1 signaling is essential for the maintenance of CSCs with EMT plasticity in pNETs through activating a partial EMT program and inducing ALDH1 and CD36 expressions. This PKD1 signaling-mediated CSC phenotype might uniquely contribute to the secondary colonization of metastatic cancer cells in other organs, such as the liver. As CD36 expression in cancer cells drives stem cell-like traits, and promotes drug resistance and metastatic potential of CSCs [17,59,62,64], PKD1-mediated CD36 expression might also play an important role in metastatic progression of pNETs, very likely via CD36-mediated fatty acid metabolism [62]. However, this concept deserves further investigation and characterization.

In addition, although PKD1 can maintain an epithelial phenotype, via negatively regulating significant molecules that regulate EMTs in some cancer cells [79], this study demonstrates that PKD1 signaling in pNETs is required for concomitant expression of vimentin and E-cadherin in CSCs. This may override the role of PKD1 in promoting the epithelial phenotype [86], leading to malignant progression and metastasis, likely by activation of a partial EMT program in CSCs [7,8,9]. Together with expression of ALDH1A1, this might render pNET cells more plastic [17,57,77] and migratory, thereby conferring high metastatic potential to CSCs.

Finally, not only does PKD1 signaling promote metastatic potential by upregulating expression of vimentin and increasing the number of ALDH1^+^ CSCs [87,88,89], this pathway may also increase the expression of CD36 to activate fatty acid metabolism and further enhance metastatic potential [62]. Therefore, identification of the PKD1 pathway in CSC plasticity with a partial EMT phenotype may provide new insights into CSC biology in a variety of cancer types, since EMT is important during the metastatic stage, while E-cadherin-induced MET facilitates subsequent colonization [42,43]. Importantly, the hybrid states of phenotypic cells may benefit the maintenance of stemness traits in pNETs [76]. It would, thus, be of interest and significance to further confirm and investigate this phenotypic change and the precise mechanisms in animal models and clinical settings. Enrichment of CSCs in the vascular niches, particularly the arteriolar niche, and CSC–EC interactions in pNET progression in vivo merits further confirmation and characterization, as the arteriolar niches have better perfusion for nutrients and oxygen to nurture CSCs. Additional animal and clinical studies may also facilitate the discovery of potential therapeutic interventions and possible biomarkers in the prediction of an unfavorable prognosis and relapse in patients with pNETs [19,49,73,90].

## 5. Conclusions

PKD1 signaling may be central in the maintenance of a unique subpopulation of CSCs, characterized by cellular plasticity and partial EMT, in pNETs. By regulating expression of cancer stemness- and EMT-related gene signatures, this pathway could promote malignant progression, metabolic reprogramming, drug resistance, and metastasis in pNETs. This deserves further elucidation. This study provides insight into the understanding of CSC-mediated malignant progression and offers vision for the development of potential therapeutic strategies in a variety of cancers with robust angiogenesis.

## Data Availability

Not applicable.

## Supplementary Materials

The following supporting information can be downloaded at: https://www.mdpi.com/article/10.3390/cells11233885/s1, Figure S1: Stem-like phenotype of cancer cells in pNETs. A. A human pancreatic tissue control (top panel) and human pNET specimens from two individual patients (middle and lower panels) were co-stained with α-SMA and CD44 antibodies followed by suitable secondary antibodies, and DAPI was used for staining the nuclei (blue). CD44-positive CSCs were marked by yellow arrowheads, which are close to the α-SMA positive (green, red arrowheads) blood vessels. The fluorescence images were acquired by an immunofluorescence microscope equipped with a CCD camera. Representative images are shown along with H & E staining for tumor tissue structures. Bar = 10 μm; Figure S2: Distribution of CD44-positive and/or PKD-1-positive CSCs in human pNET tissues. Human pNET specimens were co-stained with CD44 antibodies and PKD1 antibodies followed by suitable secondary antibodies, with DAPI staining the nuclei (blue). CD44-positive (green), PKD1-positive (red) or both positive were observed under a fluorescence microscope. Cancer cells with greater levels of both CD44 and PKD-1 (yellow arrowheads) likely left the tumor nests (white stars) and accumulated in the nearby vascular lumen (red arrowheads). Fluorescence images were acquired by an immunofluorescence microscope equipped with a CCD camera. Shown are representative images from two individual patients. Bar = 10 μm. Note: These are additional pictures for Figure 2A; Figure S3: LPA activated PKD-1 signaling pathway in pNET cells. A. Representative H & E staining images to show the pattern, shape and structure of cells in the normal pancreatic tissue samples and pNET tissues. Scale bar = 50 μm. BON cells (B) and QGP-1 cells (C) were exposed to the vehicle control, 10 μM LPA, 2 μM CRT0066101, or their combinations after 24 h. Cell lysates were extracted and the expressions of phosphorylated and total PKD-1 were detected by Western blots. Shown are the relative expression levels assessed by densitometry using an NIH Image J. Triplicate experiments were performed, and the results are shown as mean ± SEM. * *p* < 0.05, ** *p* < 0.01, and *** *p* < 0.001; Figure S4: PKD-1 on vimentin expression in pNET cells. A. QGP-1 cells were treated with 10 μM LPA, or 2 μM CRT0066101, or combination of both in medium (serum-free) for 24 h. RNA was extracted to detect mRNA levels of vimentin by RT-qPCR. B. Control and siPKD-1 transfected QGP-1 cells were treated with 10 μM LPA for 24 h. Total RNA was extracted for RT-qPCR to determine the mRNA expression of vimentin. Triplicate experiments were performed and the results were shown as the mean ± SEM. * *p* < 0.05, ** *p* < 0.01, and *** *p* < 0.001; Figure S5: Role of PKD-1 signaling in the regulation of ALDH1 activity and expression in pNET cells. A. PKD-1 siRNA transfected QGP-1 cells were incubated with antibody (ALDH1A1) followed by appropriate secondary antibody. The cells with greater levels of ALDH1A1 expression were observed and the ratio of ALHD1A1-high cells were calculated under a fluorescence microscope by counting to 100 cells randomly in each field. Fluorescence images were acquired by an immunofluorescence microscope equipped with a CCD camera, and representative images are shown. Five repetitions were performed and the statistic difference was calculated using GraphPad Prism 9. *** *p* < 0.001. B. QGP-1 cells were treated with 10 μM LPA, 2 μM CRT0066101, or their combination for 24 h. ALDH1 activity was measured by ELISA in a plate reader. Unpaired student’s *t* test was used for statistical differences. * *p* < 0.05 and ** *p* < 0.01; Table S1: Antibody Information for Western Blots (WB).
