# Supplementary material for: Protein Kinase D1 Signaling in Cancer Stem Cells with Epithelial-Mesenchymal Plasticity
Source: Cells. 2022 Dec 1;11(23):3885. doi: 10.3390/cells11233885 (PMC9739736; doi:10.3390/cells11233885)
Supplement: Supplementary file 1 [file cells-11-03885-s001.zip › cells-2008520-supplementary.pdf]

Supplementary Figures and Figure Legends

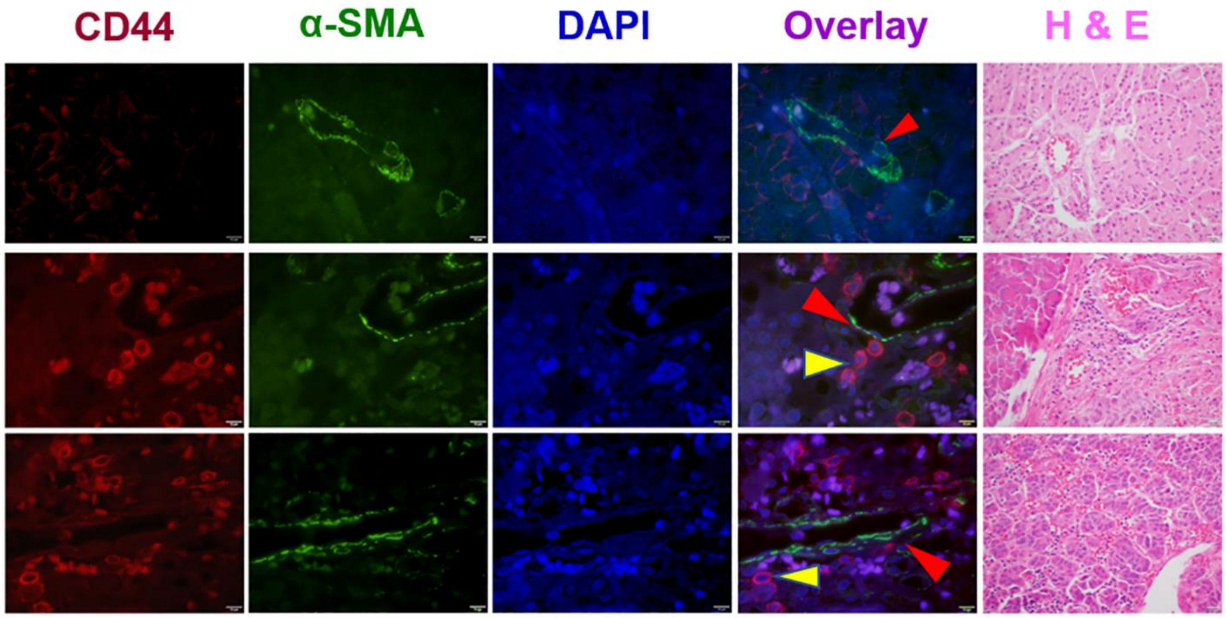

Figure S1. Stem-like phenotype of cancer cells in pNETs. **A.** A human pancreatic tissue control (top panel) and human pNET specimens from two individual patients (middle and lower panels) were co-stained with α-SMA and CD44 antibodies followed by suitable secondary antibodies, and DAPI was used for staining the nuclei (blue). CD44-positive CSCs were marked by yellow arrowheads, which are close to the α-SMA positive (green, red arrowheads) blood vessels. The fluorescence images were acquired by an immunofluorescence microscope equipped with a CCD camera. Representative images are shown along with H & E staining for tumor tissue structures. Bar = 10 μm.

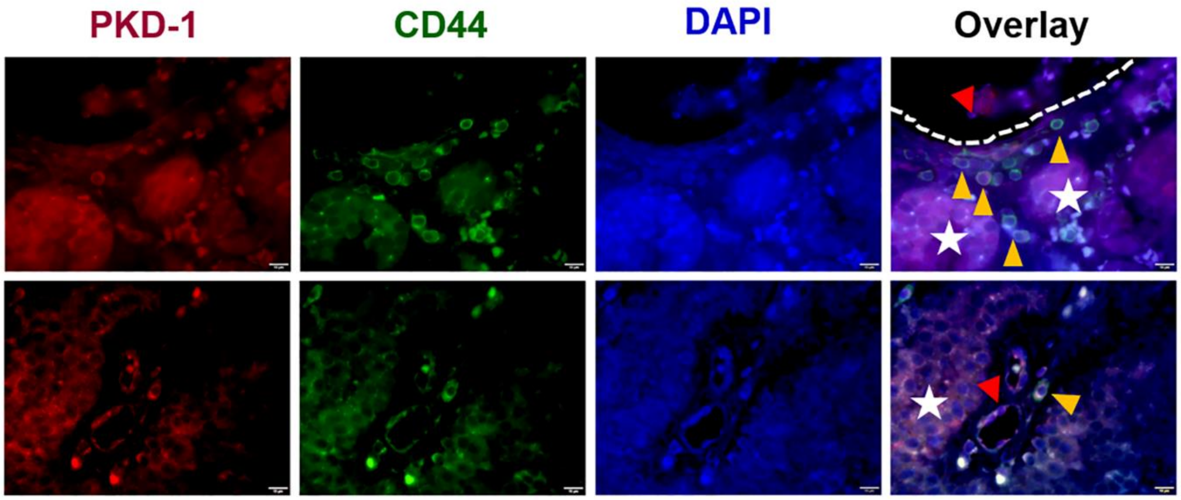

Figure S2. Distribution of CD44-positive and/or PKD-1-positive CSCs in human pNET tissues**.** Human pNET specimens were co-stained with CD44 antibodies and PKD1 antibodies followed by suitable secondary antibodies, with DAPI staining the nuclei (blue). CD44-positive (green), PKD1-positive (red) or both positive were observed under a fluorescence microscope. Cancer cells with greater levels of both CD44 and PKD-1 (yellow arrowheads) likely left the tumor nests (white stars) and accumulated in the nearby vascular lumen (red arrowheads). Fluorescence images were acquired by an immunofluorescence microscope equipped with a CCD camera. Shown are representative images from two individual patients. Bar = 10 µm. **Note:** These are additional pictures for Figure 2A.

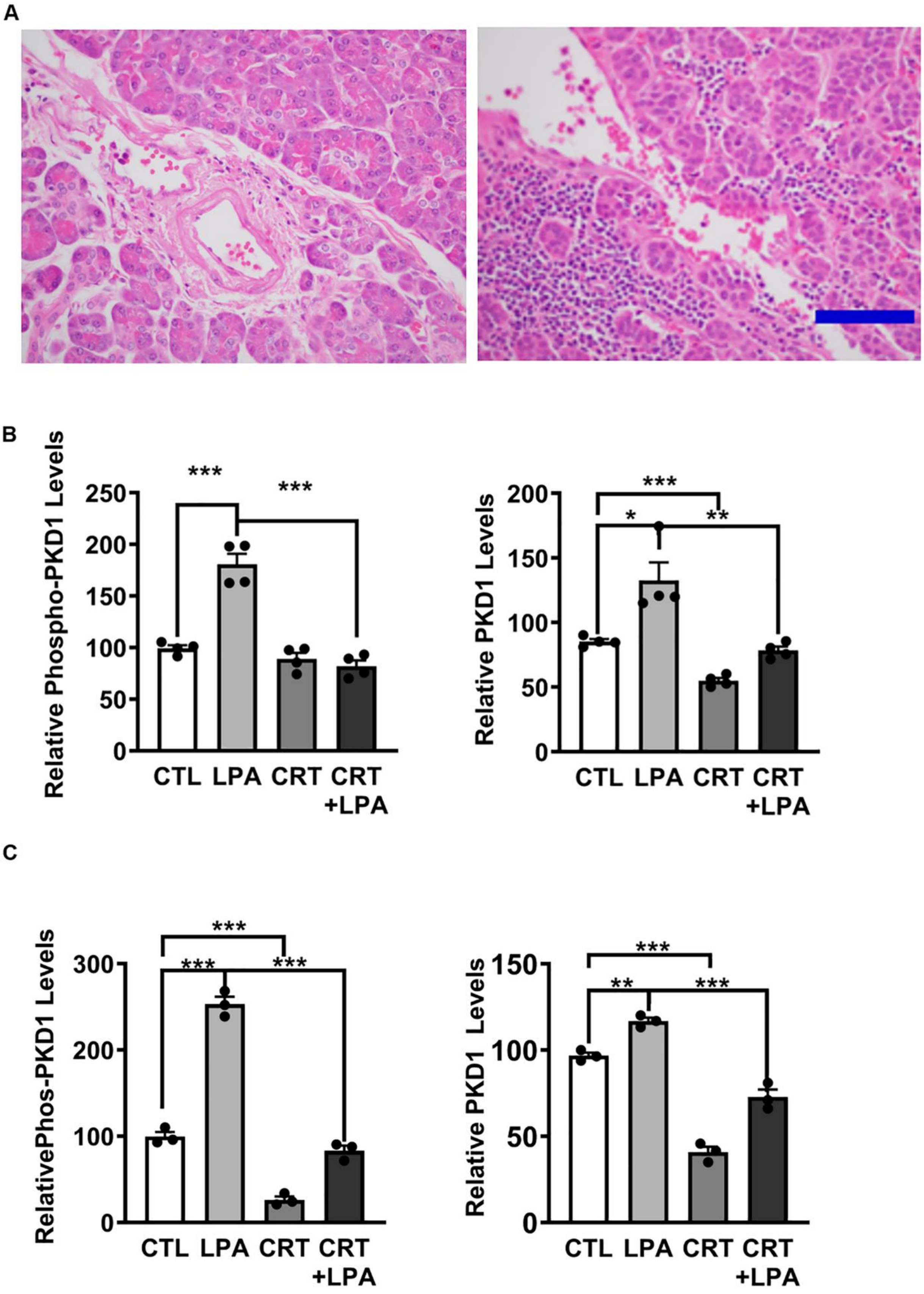
A
B
Relative Phospho-PKD1 Levels
250
200
150
100
50
0
***
***
CTL
LPA
CRT
CRT +LPA
Relative PKD1 Levels
200
150
100
50
0
***
*
**
CTL
LPA
CRT
CRT +LPA
C
RelativePhos-PKD1 Levels
300
200
100
0
***
***
***
CTL
LPA
CRT
CRT +LPA
Relative PKD1 Levels
150
100
50
0
***
**
***
CTL
LPA
CRT
CRT +LPA

Figure S3. LPA activated PKD-1 signaling pathway in pNET cells. **A**. Representative H&E staining images to show the pattern, shape and structure of cells in the normal pancreatic tissue samples and pNET tissues. Scale bar = 50 µm. BON cells (**B**) and QGP-1 cells (**C**) were exposed to the vehicle control, 10 μM LPA, 2 μM CRT0066101, or their combinations after 24 hours. Cell lysates were extracted and the expressions of phosphorylated and total PKD-1 were detected by Western blots. Shown are the relative expression levels assessed by densitometry using an NIH Image J. Triplicate experiments were performed, and the results are shown as mean ± SEM. *$P < 0.05$, **$P < 0.01$, and ***$P < 0.001$.

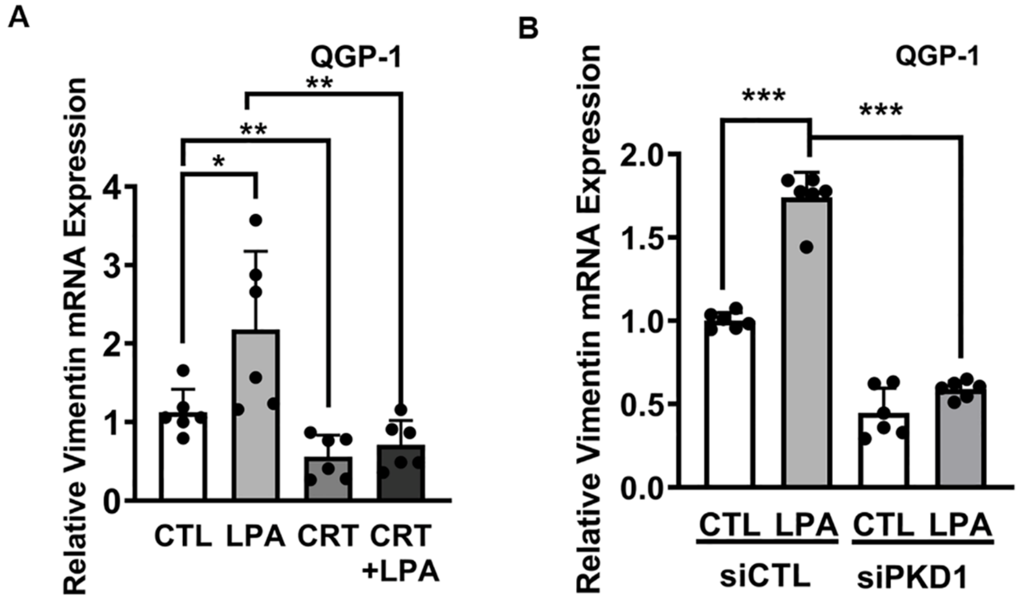

Figure S4. PKD-1 on vimentin expression in pNET cells. **A.** QGP-1 cells were treated with 10 μM LPA, or 2 μM CRT0066101, or combination of both in medium (serum-free) for 24 hours. RNA was extracted to detect mRNA levels of vimentin by RT-qPCR. **B.** Control and siPKD-1 transfected QGP-1 cells were treated with 10 μM LPA for 24 hours. Total RNA was extracted for RT-qPCR to determine the mRNA expression of vimentin. Triplicate experiments were performed and the results were shown as the mean ± SEM. *$P < 0.05$, **$P < 0.01$, and ***$P < 0.001$.

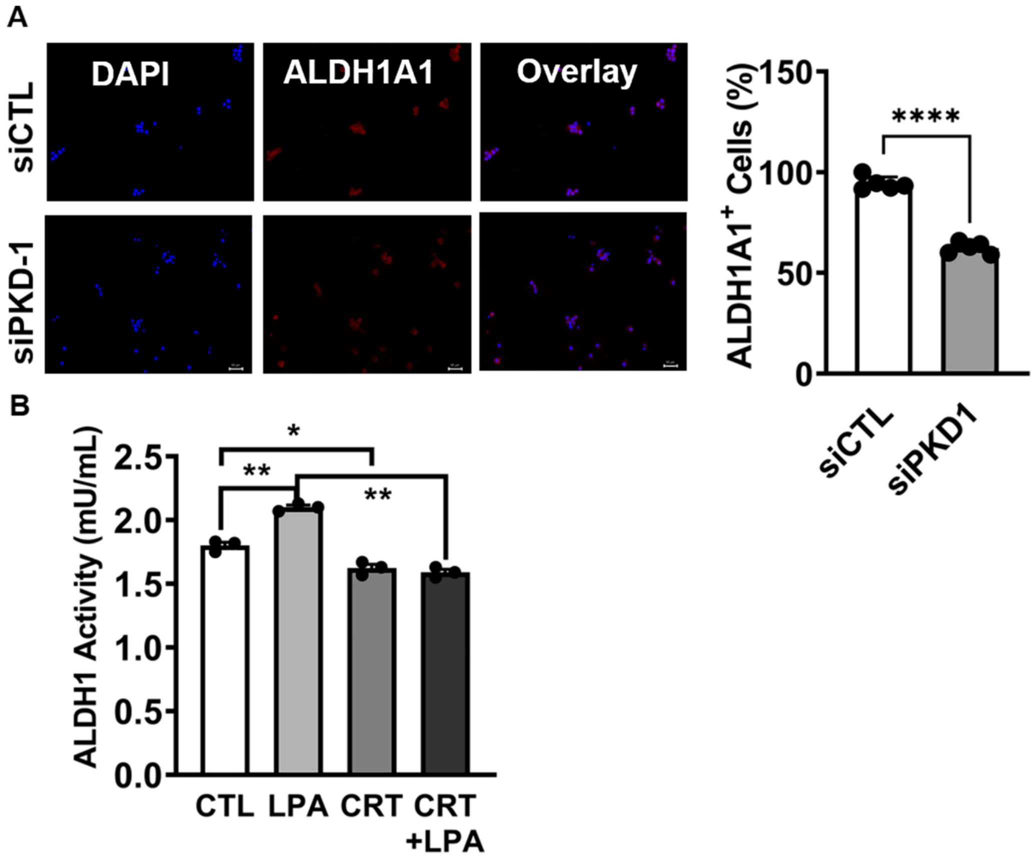

Figure S5. Role of PKD-1 signaling in the regulation of ALDH1 activity and expression in pNET cells. **A.** PKD-1 siRNA transfected QGP-1 cells were incubated with antibody (ALDH1A1) followed by appropriate secondary antibody. The cells with greater levels of ALDH1A1 expression were observed and the ratio of ALHD1A1-high cells were calculated under a fluorescence microscope by counting to 100 cells randomly in each field. Fluorescence images were acquired by an immunofluorescence microscope equipped with a CCD camera, and representative images are shown. Five repetitions were performed and the statistic difference was calculated using GraphPad Prism 9. ***$P < 0.001$. **B.** QGP-1cells were treated with 10 μM LPA, 2 μM CRT0066101, or their combination for 24 hours. ALDH1 activity was measured by ELISA in a plate reader. Unpaired student's t test was used for statistical differences. *$P < 0.05$ and **$P < 0.01$.

Table S1. Antibody Information for Western Blots (WB)

Antibody Name	Company	Catalog Number	Species	Dilution
Phospho-PKD-1	Cell Signaling	2051	rabbit	1:1000
PKD-1	Cell Signaling	90039	rabbit	1:1000
Vimentin	Cell Signaling	46173	rabbit	1:1000
β-actin	Sigma	A5441	mouse	1:5000
CD36	abcam	ab133625	rabbit	1:5000

Immunofluorescence (IF)

Antibody Name	Company	Catalog Number	Species	Dilution
CD44	Cell signaling	37259	rabbit	1:200
CD44	Cell signaling	5640	mouse	1:200
α-SMA	Sigma	A2547	mouse	1:400
CD45	Thermo	14-9457-80	mouse	1:200
PKD-1	Sigma	SAB4502371	rabbit	1:200
Vimentin	Cell Signaling	5741	rabbit	1:200
ALDH1A1	R&D Systems	AF5869	goat	1:200

Immunohistochemistry (IHC)

Antibody Name	Company	Catalog Number	Species	Dilution
E-Cadherin	Cell signaling	3195	rabbit	1:200
N-Cadherin	Cell signaling	13116	rabbit	1:200
Vimentin	Cell signaling	5741	rabbit	1:200
